# Association between general and oral health-related quality of life in patients treated for oral cancer

**DOI:** 10.4317/medoral.20714

**Published:** 2015-10-09

**Authors:** Rocío Barrios, Georgios Tsakos, José-Antonio Gil-Montoya, Javier Montero, Manuel Bravo

**Affiliations:** 1 PhD, Postgraduate Research Fellow of the Spanish Ministry of Education; 2 PhD, Senior Lecturer and Honorary Consultant in Dental Public Health; 3 PhD, Associated Professor of Special Care in Dentistry and Gerodontology; 4 PhD, Associated Professor of Prosthetic Dentistry; 5 PhD, Professor of Preventive and Community Dentistry

## Abstract

**Background:**

Less is known about the association between general health-related quality of life (HRQoL) and oral HRQoL (OHRQoL) among patients with specific diseases. The aim of this study was to assess the association between patient-centered outcome measurements (HRQoL and OHRQoL) of oral cancer patients at least 6 months after treatment.

**Material and Methods:**

HRQoL was measured with the 12-Item Short Form Health Survey (SF-12); OHRQoL was evaluated using the Oral Health Impact Profile (OHIP-14) and the Oral Impacts on Daily Performances (OIDP).

**Results:**

Higher OHRQoL scores were associated with lower SF-12 domains scores. The OHIP-14 explained 16.5 % of the total variance of SF-12 Physical Component Summary (PCS) and the OIDP explained 16.1 %. In the SF-12 Mental Component Summary (MCS), the total variance explained was 23.9 % by the OHIP-14 and 21.8 % by the OIDP.

**Conclusions:**

There was a significant association between long-term OHRQoL and HRQoL in oral and oropharyngeal cancer patients. These results may help to carry out new interventions aiming to improve patient´s life overall.

**Key words:**Mouth neoplasms, quality of life, health status, oral health.

## Introduction

There has been an increase in the use of health-related quality of life (HRQoL) measures to describe the outcomes of health conditions and the effectiveness of their treatment. This concept is based on the perception of the individual (1) and helps to understand the impact of conditions, assesses the effect of their treatment on reducing the disease burden, and provides an insight into which parameters are perceived as most important (2).

Similarly, oral health-related quality of life (OHRQoL) focuses on the subject’s perception about how his/her oral health affects the quality of life. So, it would be logical to assume that OHRQoL is related to HRQoL, however, the links between OHRQoL and life overall should be demonstrated and not merely assumed (3). One possible way would be the concurrent use of OHRQoL measures with global ratings of general HRQoL since it would improve the understanding of the consequences of oral disorders (4).

HRQoL and OHRQoL have been shown to be associated in the general population (5) but this association may differ among patients with specific diseases. Head and neck cancer and the side-effects of the treatment have negative impact on many different aspects of quality of life patients over time (6). Oral cancer patients are considered different to patients suffering from other head and neck tumours because of the complex tridimensional anatomy of the mouth. Therefore, the use of site-specific analysis of outcomes, within the head and neck region, has been recommended (7).

A recent review has been done about studies that looked at the HRQoL and OHRQoL of patients treated for oral and oropharyngeal cancer (8). However, little is known about the relationship between HRQoL and OHRQoL, because their findings of the studies were inconclusive. Most of them found positive correlations between HRQoL and OHRQoL (9-11) but Pierre *et al*. (12), did not find any correlation. Another way to address this relationship would be to use generic scales in order to include items that cover all major aspects of the person’s health (13).

Therefore, the aim of this study was to assess the association between long-term OHRQoL and HRQoL of a homogeneous group of oral and oropharyngeal cancer patients at least 6 months after treatment, in Granada, Spain.

## Material and Methods

- Patients

This study is part of a larger project about factors associated to the HRQoL in patients treated for oral cancer (14). This study was carried out at the Department of Maxillofacial Surgery of the Virgen de las Nieves University Hospital of Granada. Inclusion criteria for participation in the study were: patients treated for oral or oropharyngeal cancer, at least six months have elapsed since treatment and the patients were free from recurrence of the disease. Exclusion criteria were: patients treated for other type of cancer or patients with inability to complete or respond to questionnaires. A total of 145 cases fulfilled the inclusion/exclusion criteria and were initially selected. Of them, 3 cases did not accept to participate in the study, giving 142 cases (97.9% acceptance rate) for the analysis.

As explained above, this study is part of a larger project (142 oral cancers and 142 control patients) (14). The initial sample size was established to detect a 0.3 standardized difference between cases and controls for the outcome variables (OHRQoL). For this present study, with only 142 cases and with the main aim of studying the association between OHRQoL and HRQoL, a sample size of 142 allows to detect as statistically significant (alpha=0.05), and according to Sample Power 2.0 (SPSS Inc., Chicago, IL), a relatively small correlation of 0.23, with 80% power (beta=0.20).

The study was approved by the Ethics Committee of the University of Granada and each participant signed an informed consent.

Collected data included HRQoL as the outcome variable, OHRQoL as the main exposure variable and sex, age, social class, tumor site, clinical stage, follow-up, type of treatment, and presence of chronic diseases as covariates. The chronic diseases present were: diabetes, hypertension, high cholesterol levels, coronary heart diseases and lung diseases.

- Measurement of HRQoL

The 12-Item Short Form Health Survey (SF-12) was used to evaluate HRQoL. The SF-12 is a shorter version of one the most commonly used general questionnaire (15), the 36-Item Short Form Health Survey (SF-36) (16). The version 2 of SF-12 permits the calculation of the 8 original SF-36 dimensions scores while version 1 does not (17,18). This validated instrument (19) contains 12 ítems with 3- or 5-point Likert scales resulting in 8 dimensions: physical functioning, role physical, bodily pain, general health, vitality, social functioning, role emotional and mental health. Physical Component Summary and Mental Component Summary are two summary scores calculated from these dimensions.

To compute SF-12 scores (19), first, we calculated the scores of the 8 dimensions and transformed them to a 1-100 scale; then, we standardized them and finally did a linear transformation (multiplying the scores by 10 and adding 50) to obtain a distribution of mean of 50 and standard deviation of 10 in the reference general population. Computations of the aggregate summary components consist of multiplying the standardization by its respective physical or mental factor score coefficient and summing the eight products. The last step also involves transforming the aggregate physical and mental summary scores to the norm-based {50,10} scoring. We chose this specific method for the calculation using the SF-12 reference standards for the Spanish population (17). Higher scores indicate better quality of life.

- Measurement of OHRQoL

OHRQoL was assessed through two widely used relevant generic measures: The Oral Health Impact Profile (OHIP-14) and the Oral Impacts on Daily Performances (OIDP).

The OHIP-14 contains 14 items that are grouped into seven dimensions of impact: functional limitation, physical pain, psychological discomfort, physical disability, psychological disability, social disability and handicap. The participants respond to each item according to the frequency of the impact on a 5-point Likert scale (ranging from 0 to 4): never, hardly ever, occasionally, fairly often, and very often (20). The additive method (OHIP-AD) scoring method was used where the dimensions and the total score were calculated by summing the number of impacts reported.

The OIDP assesses the impact of oral conditions on eight daily life performances: eating, speaking, cleaning teeth, carrying out major work or role, social contact, relaxing/sleeping, smiling, and emotional state. It takes into account the frequency and the severity of these impacts through Likert scales. For each performance a score is calculated by multiplying the frequency and severity scores. To get a percentage overall score, the sum of these performances scores is divided by the maximum possible score and multiplied by 100 (21,22).

For both the OHIP-14 and the OIDP, a higher score indicates worse OHRQoL. The participants were interviewed at least 6 months after the end of their oral cancer treatment. In order to avoid including the acute period of recovery in the time frame in cases of recent treatment the recall period for both OHIP-14 and OIDP was changed from the usual 12 or 6 months to 1 month.

- Statistical analysis

Statistical analysis was performed using the SPSS version 17.0 software package (SPSS Inc., Chicago, IL). Descriptive analysis of socio-demographic and clinical variables, SF-12, OHIP-14 and OIDP was followed by bivariate associations between the covariates and the SF-12 domains using the appropriate test according to the type of variable (Pearson’s or Spearman’s correlation test).

Linear regression models were carried out to evaluate the variance in SF-12 summary components (Physical Component Summary score, Mental Component Summary score) by covariates registered (potential confounding factors). The two OHRQoL measures were used interchangeably in the models (they were tested one at a time in each multiple regression model). Model 1 was built by introducing age, sex and the statistically significant variables from the respective bivariate analysis (chronic diseases). It assessed the adjusted association between the outcome (SF-12 summary components) and each one of the main exposures. Model 2 was built with the backward regression method to select the best model to predict SF-12 Physical Component Summary score and SF-12 Mental Component Summary from the different exposures. The level of significance for the removal of variables was set at *p* > 0.10. The coefficients of determination (r2) of the models were calculated. Models were run separately for the Physical Component Summary score and the Mental Component Summary score.

Authors have followed the STROBE guidelines for carrying out the study and for writing the paper (23).

## Results

The social and clinical profile of the sample is presented in  Table 1. Of 142 patients, 91 were males (64.1 %), with a mean age of 65.2 (standard deviation: 12.9) years. The lowest social class (V) was the most frequent in our sample. The tongue was the most frequent location for the cancer, the clinical stages I and IV the most prevalent, the mean follow-up was 4.0 (standard deviation: 4.3) years and most patients underwent surgery without radiotherapy or chemotherapy.

**Table 1:**
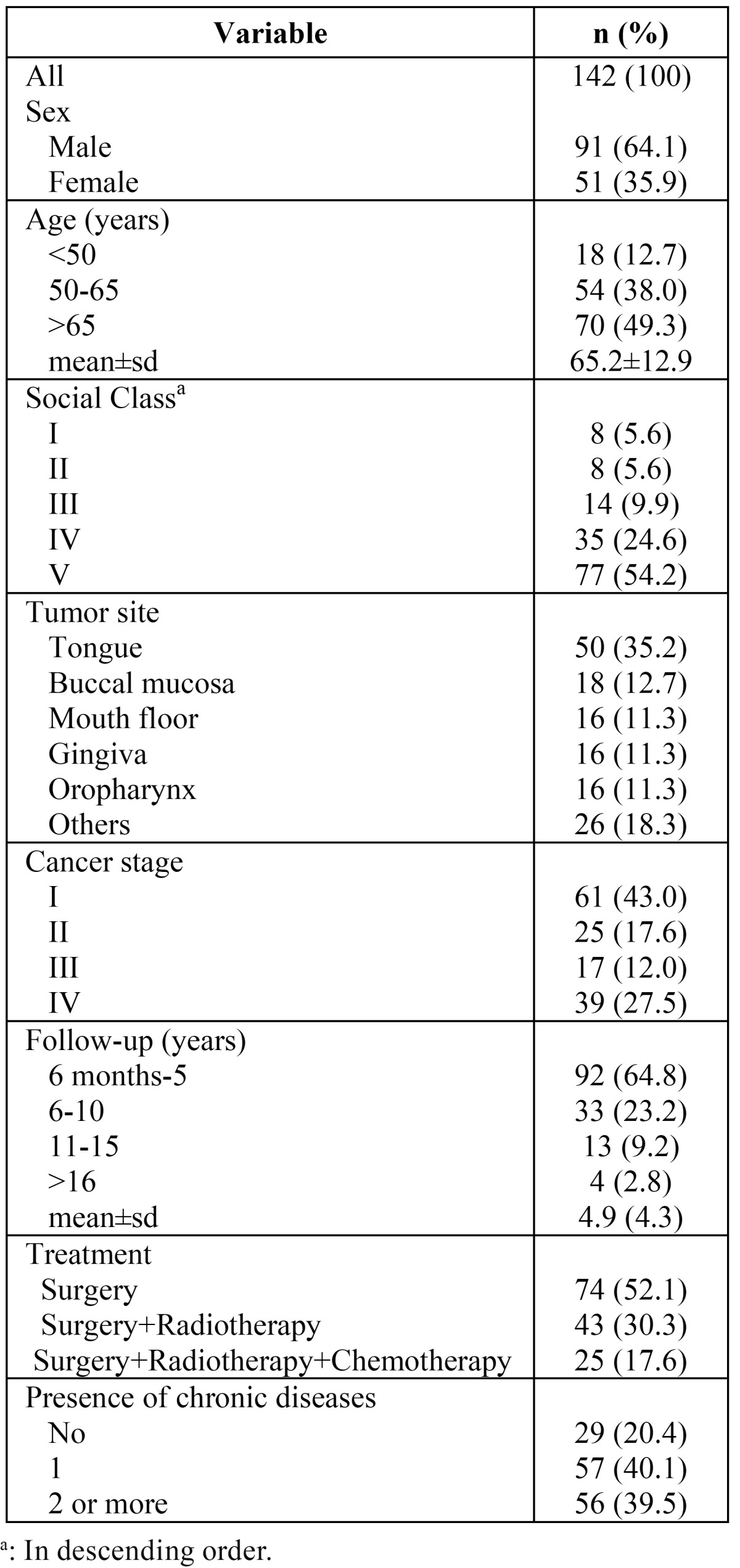
Socio-demographic and clinical data variables description of oral cancer patients (n=142).

In relation to the HRQoL, the role physical and physical functioning were the SF-12 domains with worse scores. For OHRQoL, oral impacts predominantly affected the OHIP-14 dimensions on physical pain, physical disability and functional limitation while for the OIDP the items on difficulty eating, difficulty speaking and maintaining emotional status were the most prevalent ( Table 2).

**Table 2:**
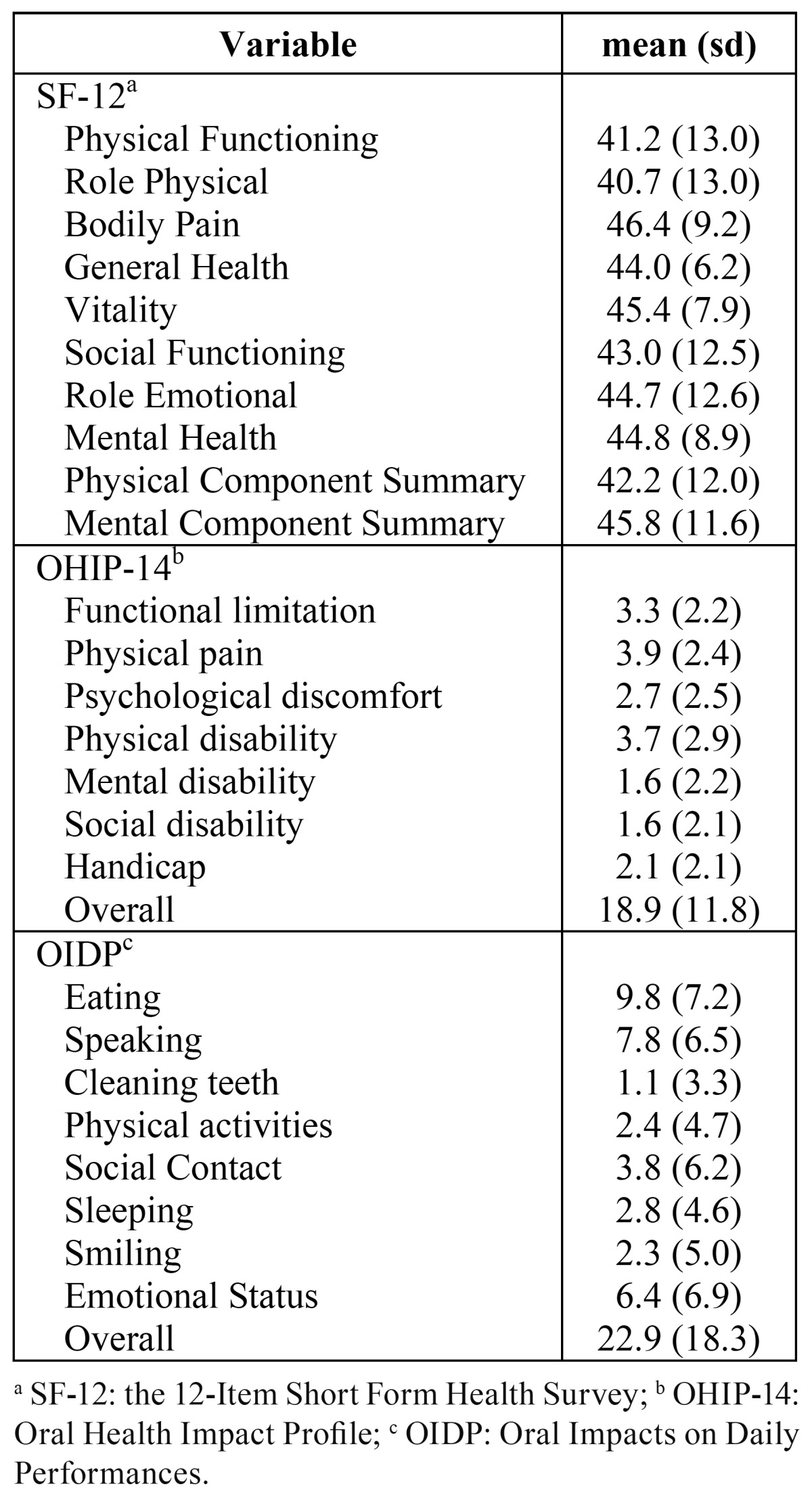
Health-related quality of life (SF-12) and oral health-related quality of life (OHIP-14 and OIDP) in oral cancer patients (n=142).

The OHIP-14 and the OIDP were significantly correlated with all domains and summary components of the SF-12. Being female and having chronic diseases were correlated with worse scores in all domains of SF-12, with most of these associations being statistically significant (physical functioning, role physical, bodily pain, general health, vitality, mental health and Physical Component Summary with respect to sex and bodily pain, general health, vitality, social functioning and Physical Component Summary with respect to chronic diseases). There were significant negative correlations between age and all SF-12 domains, being statistically significant in physical functioning, role physical, vitality, social functioning and physical component summary. Neither social class nor clinical variables were significantly correlated with SF-12 (data not shown).

 Table 3 shows multiple linear regression analyses. Higher OHIP-14 and OIDP scores were associated with lower SF-12 scores. In adjusted analyses, for each higher unit in OHIP-14 or OIDP score, the Physical Component Summary (PCS) was lower; the regression coefficients were -0.22 [-0.38-(-0.06)] units and -0.21 [-0.24-(-0.03)] units respectively. After applying backward regression method (Model 2) age and sex were the only other variables that contributed to the models for PCS. There was hardly any change in the crude estimates: the coefficients were -0.22 [-0.38-(-0.07)] for the OHIP-14 and -0.21 [-0.24-(-0.04)] for the OIDP. For the association between OHRQoL and the MCS SF-12 component, lower MCS scores were associated with 1 higher unit in the OHRQoL measures, with the respective coefficients being -0.50 [-0.64-(-0.34)] for the OHIP-14 and -0.45 [-0.40-(-0.21)] for the OIDP. Following the same procedure (backward regression method) to identify significant predictors for the MCS of the SF-12, only the OHRQoL variables remained in the models (Model 2). The inclusion of none of the other variables could improve the predictive ability of the models.

**Table 3:**
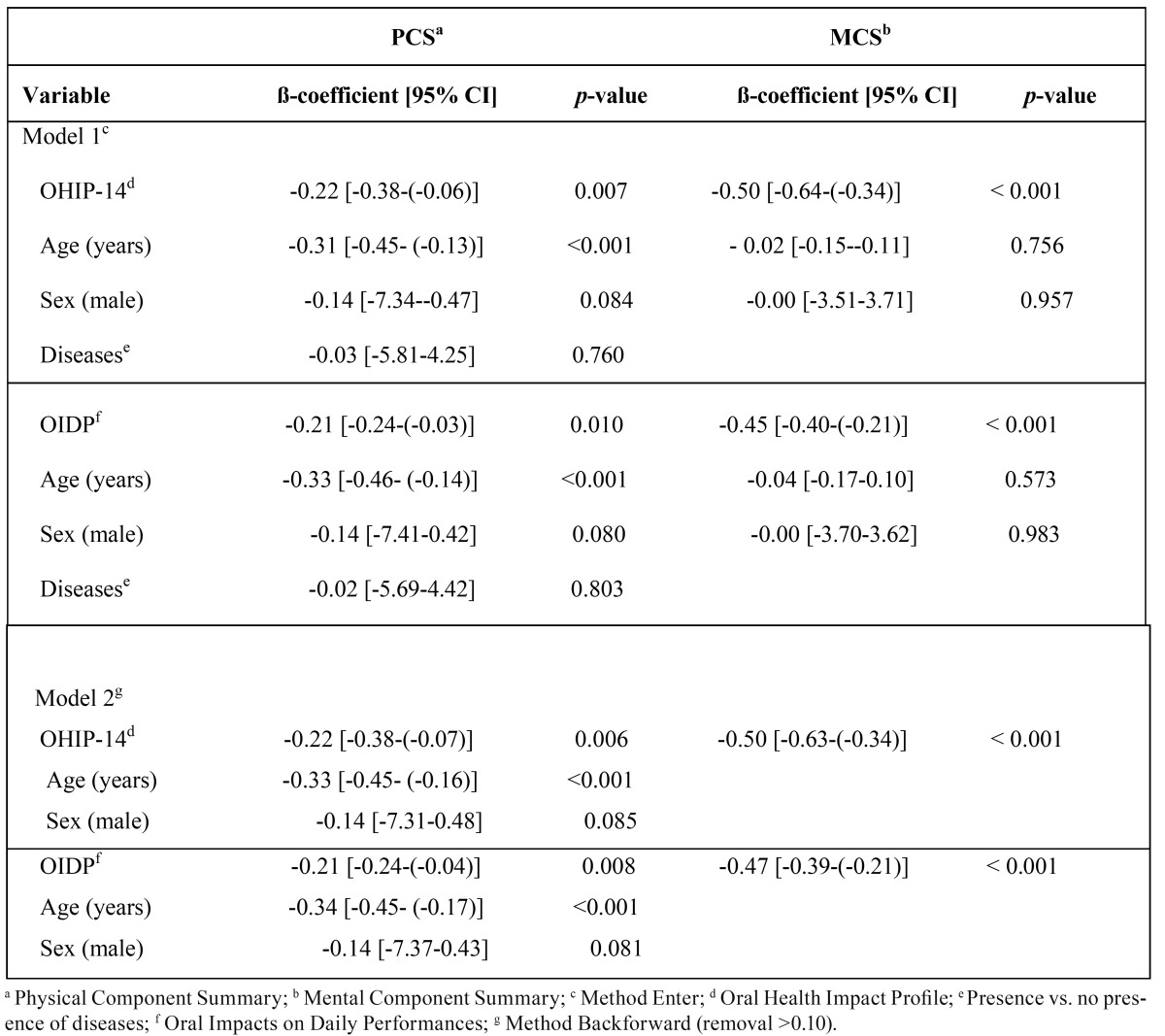
Multiple linear regression models predicting SF-12 Physical Component Summary and SF-12 Mental Component Summary scores in patients treated for oral cancer (n=142).

According to the coefficient of determination (r2), the final model for the OHIP-14 explains 16.5% of the total variance of PCS and the respective figure for the model with the OIDP was 16.1%. In terms of the MCS, the total variance explained was 23.9% for the model with the OHIP-14 and 21.8% for the model with the OIDP.

## Discussion

This study showed a significant association between long-term OHRQoL and HRQoL in oral and oropharyngeal cancer patients after at least 6 months post-treatment. This relationship remained significant after adjusting for significant covariates. Moreover, when looking for the best predictive model for the PCS and the MCS, OHRQoL measures were implicated in both models and were the only variables that remained in the final models for the MCS.

We found a significant association between OHRQoL and all SF-12 domains. This finding is in line with previous studies that showed correlations between global HRQoL and some head and neck cancer symptoms (9-11) or with the head and neck cancer symptom scale (10). Pierre *et al*. (12), however, did not find such an association. Differences in the treatment regimen, the follow-up time or the questionnaires used for evaluation hinder the interpretation and comparison of published results on HRQoL and OHRQoL of oral cancer patients. We chose the two more widely used generic instruments to evaluate the OHRQoL which could minimize the chance that the observed associations are due to the selected questionnaire to assess OHRQoL.

OHRQoL was strongly correlated to SF-12 MCS with worse OHRQoL being associated with worse SF-12 MCS levels. This association may indicate that oral impacts may be of sufficient magnitude or duration to compromise the overall quality of life. Previous literature has shown that OHRQoL was significantly correlated with psychological factors, such as depression and anxiety (24). On the other hand, another study suggested that patients view their oral health as impaired only if the symptoms of disease affect their functioning. In the same way, it is suggested that the functioning of the mouth or body could be seen as a link between HRQoL and OHRQoL (25). Our findings somehow support this point since we found a significant association between OHRQoL and SF-12 domains, with physical functioning being one of the most impaired SF-12 domains and the same was the case for OHIP-14 (functional limitation) and OIDP (difficulty eating and difficulty speaking).

Multivariate models may facilitate understanding of the factors that influence HRQoL and therefore are potentially useful for the development of interventions aiming to improve HRQoL (13). Although socioeconomic status is a well-known predictor of disease morbidity or mortality rates, there is controversy about its association with HRQoL in the literature (26,27). In our study, social class was not associated with HRQoL. As in previous studies (10,28), clinical variables such as clinical stage, follow-up or type of treatment were not significantly associated with HRQoL in bivariate analyses. This could be partly due to the fact that our patients were evaluated at least 6 months after treatment. First, differences in HRQoL for clinical stage tend to be minimized over time (15). Second, the impairments in oral cancer patients can last a long time which would explain that more time of follow-up was not correlated to worse HRQoL. Finally, differences in HRQoL according to type of treatment are related to oral symptoms (15), that it is to say, they are more related to OHRQoL than HRQoL.

OHRQoL was the only variable to be associated to both HRQoL components and was the only variable in the MCS model. Furthermore, it is noteworthy that OHRQoL accounted for a considerable percentage of the total variance in the MCS (23.9% for OHIP and 21.8% for OIDP). For the PCS, OHRQoL, age and sex explained 16.5% of the total variance when the OHIP-14 was used and 16.1% when the OIDP was used.

In line with other studies (7,27,29) older patients had significantly worse HRQoL scores a long-time after treatment than younger patients. There is incongruence in the literature regarding the role of gender in determining HRQOL (29) we found that women had worse PCS scores than men. Age and sex are biological characteristics that may help identify patients most likely to benefit from supportive care options. From a public health point of view, it is important to highlight that OHRQoL is the only variable in the multivariate models that it is amenable to modification though interventions. The negative significant association between OHRQoL and both PCS and MCS indicates that focusing on factors that might positively influence OHRQoL could potentially contribute to improving HRQoL.

This study has some limitations. A first potential limitation related to the use of nonspecific disease questionnaires. Because oral cancer is such a disabling condition, it is quite likely that the vast majority of the reported oral impacts would relate to this condition or its treatment. This could have been addressed through the additional use of a cancer-specific OHRQoL measure. The OIDP has a condition specific feature that attributes oral impacts to specific conditions (30), oral cancer in this case. It was not used in this study to avoid increasing data collection time and reduce the efficiency of the study. Nevertheless, generic questionnaires are beneficial in other respects as they are conceptually broader and cover large aspects of a person’s health (13). Second, we did not have information on other factors that may also be influence HRQoL such as marital status or behaviors like alcohol or smoking. Future research should include cancer-specific assessment of OHRQoL and consider other factors that could be associated with the change in long-term HRQoL in oral and oropharyngeal cancer patients.

In conclusion, There was a significant association between long-term OHRQoL and HRQoL in oral and oropharyngeal cancer patients, ever after adjusting for confounding factors. OHRQoL could be an important factor determining HRQoL, particularly SF-12 MCS.
